# External Cephalic Version—A Chance for Vaginal Delivery at Breech Presentation

**DOI:** 10.3390/medicina58111619

**Published:** 2022-11-10

**Authors:** Ionut Marcel Cobec, Vlad Bogdan Varzaru, Tamas Kövendy, Lorant Kuban, Anca-Elena Eftenoiu, Aurica Elisabeta Moatar, Andreas Rempen

**Affiliations:** 1 Clinic of Obstetrics and Gynecology, Diakoneo Diak Klinikum Schwäbisch Hall, Diakoniestrasse 10, 74523 Schwäbisch Hall, Germany; 2 Clinic of Internal Medicine, Hohenloher Krankenhaus Öhringen, 74613 Öhringen, Germany

**Keywords:** external cephalic version, breech presentation, caesarean section, vaginal birth

## Abstract

*Background and Objectives*: In recent years, the rate of caesarean section (CS) has increased constantly. Although vaginal breech delivery has a long history, breech presentation has become the third most common indication for CS. This study aims to identify factors associated with the success of external cephalic version (ECV), underline the success rate of ECV for breech presentation and highlight the high rate of vaginal delivery after successful ECV. *Material and Methods*: This retrospective observational study included 113 patients with singleton fetuses in breech presentation, who underwent ECV from January 2016 to March 2021 in the Clinic of Obstetrics and Gynecology, Diakonieklinikum Schwäbisch Hall, Germany. Maternal and fetal parameters and data related to procedure and delivery were collected. Possible predictors of successful ECV were evaluated. *Results*: The success rate of ECV was 54.9%. The overall rate of vaginal birth was 44.2%, regardless of ECV outcome. The vaginal birth rate after successful ECV was 80.6%. Overall, 79.0% of women with successful ECV delivered spontaneously without complications, 19.4% delivered through CS performed during labor by medical necessity, and 1.6% delivered through vacuum extraction. ECV was performed successfully in three of the four women with history of CS. Gravidity, parity, maternal age, gestational age, fetal weight, and amniotic fluid index (AFI) were significantly correlated with the outcome of ECV. *Conclusions*: ECV for breech presentation is a safe procedure with a good success rate, thus increasing the proportion of vaginal births. Maternal and fetal parameters can be used to estimate the chances of successful ECV.

## 1. Introduction

In recent years, the rate of caesarean section (CS) has increased constantly in Germany [1]. In singleton pregnancies, an important indication of CS has been fetal malpresentation. In clinical practice, breech presentation (praesentation caudae) is the most common abnormal fetal presentation, which refers to fetuses lying bottom- or feet/knee-first rather than head-first [2]. Breech presentation is defined as a longitudinal positioning of the fetus with the buttocks or feet closest to the cervix. In Germany, fetal breech presentation at term occurs in about 3% of singleton pregnancies. The rate of breech presentation decreases with gestational age. This rate is about 9% between 33 and 36 pregnancy weeks, 18% between 28 and 32 weeks, and about 30% before the 28th pregnancy week [3]. 

The predisposing factors for breech presentation are uterine anomalies (e.g., uterus arcuatus, uterus bicornis, uterus duplex), uterus myomatosus, pelvic tumor, advanced multiparity, history of cesarean delivery or breech delivery, gestational diabetes, multiple gestation, congenital anomalies of the fetus (neural tube defects, fetal hydrocephalus or anencephaly), neuromuscular diseases, cephalo-pelvic disproportion, prematurity, low fetal birth weight, oligohydramnios, short umbilical cord, polar placentation, and placenta praevia [4,5]. However, in about 75% of cases, no specific cause of term breech presentation could be identified [4,6]. The main types of breech presentation are frank (≈60–70%), complete (≈4–10%), and incomplete breech (≈20–36%) [7,8]. 

Vaginal breech delivery has a long history. Studies have shown that perinatal and neonatal mortality rates, as well as serious neonatal morbidity rates, were higher in the planned vaginal delivery than in the planned cesarean delivery at breech presentation [9]. These findings significantly lead to CS being accepted by obstetricians as the safer option for breech delivery [9].

In the United States, there has been an increase in the frequency of CS in the past 20 years. One in three women giving birth in the USA will undergo a CS [10]. In many other developed and developing countries, this rate is the same. For example, in Korea, the frequency of CS was about 36.9% in 2012, CS being the usual method of delivery for term breech presentation [11]. Breech presentation became the third most common indication for CS, after previous CS and labor dystocia [12]. 

The maternal morbidity of CS is approximately three times higher than that of vaginal delivery [13]. The maternal risks of CS compared to vaginal delivery are well known. These include greater blood loss, thrombotic events, unplanned hysterectomy, operative damage to other organs, mortality, longer hospital stay with higher costs, and more readmissions than patients undergoing vaginal delivery [14]. Additional maternal complications of CS include scarring, chronic pain, and intestinal obstruction caused by adhesive disease. Moreover, in the following pregnancies, a previous cesarean delivery may cause a higher rate of placental abnormalities, unexplained stillbirth, as well as repeated surgical delivery in many cases [14]. However, vaginal delivery could also have maternal complications compared to CS, such as postpartum urinary incontinence and pelvic organ prolapse [15].

In case of fetal breech position, the external cephalic version (ECV) could be an option for reducing the number of CSs and vaginal breech deliveries [9]. ECV is a technique used to convert the fetal breech presentation into a cephalic position with targeted manual pressure on the mother’s abdominal wall at-term or near-term pregnancies in order to increase the chance of a vaginal cephalic birth [9,16,17]. ECV can be carried out with or without analgesics and with or without tocolytic therapy [18]. 

Factors favoring the success of ECV could be multiparous women, non-anterior placental location, palpability of the fetal skull, lower maternal body mass index, the type of breech presentation (for example, the frank breech presentation is associated with lower rates of success) and, of course, the experience of the physician in performing ECV [10,18,19]. Placental abruption, vaginal bleeding, fetal injury (including fractures and brachial plexus injuries), and pathological cardiotocography (CTG) findings, such as fetal bradycardia, may represent complications of the method [20]. 

The aim of this study is to identify factors associated with the success of ECV, highlight the relevance and success rate of ECV for breech presentation, and underline the high rate of vaginal deliveries in patients with successful ECV for breech presentation.

## 2. Material and Methods

This study represents a retrospective and anonymized data analysis over a period of 5 years. We reviewed the records of 113 women who underwent ECV from January 2016 to March 2021 in the Clinic of Obstetrics and Gynecology, Diakoneo Diak Klinikum Schwäbisch Hall, Germany. In our study, we included all patients with singleton fetuses in breech presentation who agreed to the maneuver. The ECV was performed by different senior consultants. Prior to ECV, an ultrasound control was performed, and the possible risks of the maneuver were discussed. Each patient signed the ECV informed consent. ECV was not performed if the patient rejected ECV or if there were absolute contraindications of ECV.

For 30 min before and during the ECV, the patient received an infusion with tocolysis with fenoterol. Before and after the ECV, a CTG control was performed. The ECV was attempted under ultrasound control of the fetal heartbeat. Fetal biometric parameters were obtained sonographically. The patient was placed in a comfortable lying position with knees slightly elevated. The patient was allowed to end the maneuver at any point in time.

Maternal age, number of pregnancies, number of childbirths, history of CS, ultrasonographic findings (type of breech presentation, placental location, amniotic fluid index), characteristics of ECV (gestational age at ECV, fetal weight at ECV, success of ECV, direction in which successful ECV was performed, complications during and after ECV), and birth-related characteristics (planned and real type of delivery, gestational age at birth, fetal weight at birth) were collected from our database. Data were analyzed using IBM SPSS Statistics 20. Grouping by the dichotomous outcome of ECV, we used either *χ*^2^ analysis or Fisher’s exact test for categorical variables and independent samples *t*-test for continuous variables. Multiple binary logistic regression was used to identify possible predictors of the outcome of ECV. We used the significance threshold of α = 0.05 corresponding to the 95% confidence interval.

## 3. Results

In the observed five years, we registered 6619 singleton deliveries out of a total of 6825 deliveries and a general CS rate of 24.9%. Overall, 11.0% were elective CSs and 13.9% CSs were performed during labor by medical necessity. In total, 4.8% of all registered deliveries in our clinic in the observed period were CSs with breech presentation. In our sample of 113 women, the mean maternal age was 31.69 years (*SD* = 4.44)—the youngest patient was 18 years old and the oldest patient was 43 years old. In total, 53.1% of the women were primigravida and 61.9% were nullipara. Four (3.5%) women had a history of CS. 

Before ECV was performed, the fetal back faced the maternal left in 60 (53.1%) cases and the maternal right in 53 (46.9%) cases. In 56 (49.6%) cases, the placenta was located on the posterior wall, in 47 (41.6%) on the anterior wall, in 6 (5.3%) in the fundus, and in 4 (3.6%) on the left or right wall. The mean amniotic fluid index (AFI) at ECV was 14.88 (*SD* = 3.58), ranging from 8 to 25. The mean gestational age at ECV was 261.82 days (*SD* = 4.98). The minimum gestational age at ECV in our cohort was 35 + 2 weeks of pregnancy and the latest performed ECV was at 40 + 0 weeks of pregnancy. In 12 cases (10.6%), ECV was performed under 37 weeks of gestation because of medical necessity and with informed patient consent. The mean fetal weight at ECV was 2966.02 g (*SD* = 391.06), ranging from 2158 g to 4123 g. 

The success rate of ECV was 54.9%. ECV succeeded backwards in 39 (62.9%) cases and forwards in 23 (37.1%) cases. Overall, 101 (89.4%) of the ECVs were performed without any complications during the maneuver. In total, 12 (10.6%) cases encountered complications during the attempt of ECV. The complications were represented by fetal bradycardia with quick recovery in 7 cases, maternal intolerable abdominal pain in 2 cases, vena cava compression with quick recovery in 1 case, low maternal tocolysis tolerance in 1 case, and maternal nausea and emesis in 1 case. A single patient (0.9%) developed contractions during post-ECV monitoring, while 112 patients (99.1%) had no complications post-ECV. 

The overall rate of vaginal birth was 44.2%, regardless of ECV outcome. The successful ECV group was planned for spontaneous delivery. The vaginal birth rate of the successful ECV group was 80.6%. Out of 62 patients, 49 (79.0%) delivered spontaneously without complications, 12 (19.4%) delivered through CS performed during labor by medical necessity, and 1 (1.6%) delivered through vacuum extraction. ECV was performed successfully in three of the four women with history of CS; three delivered through CS and one delivered vaginally. The unsuccessful ECV group delivered through CS.

For gestational age and fetal weight at birth, eight observations were excluded from the analysis due to missing values. Five patients were planned for CS and decided to deliver in another clinic, while three patients were planned for spontaneous delivery and decided upon home birth. The mean gestational age at birth was 275.41 days (*SD* = 8.96), the earliest delivery was at 37 + 0 weeks of pregnancy and the latest was at 42 + 0 weeks of pregnancy. The mean fetal weight at birth was 3350.43 (*SD* = 470.69), ranging from 2180 g to 4470 g.

We analyzed the relationship between the outcome of ECV and the following categorical variables: gravidity, parity, history of CS, fetal back position before ECV and placental location (Table 1). Multigravidity, defined as having been pregnant more than once, and a parity ≥ 1 were significantly associated with a successful ECV.

We compared maternal age, gestational age, fetal weight and AFI at ECV for successful and unsuccessful ECV using an independent samples *t*-test and found significant differences (Table 2). For gestational age, we conducted a Welch’s *t*-test since equal variances could not be assumed. The other continuous variables were compared using Student’s *t*-test.

Multiple logistic regression analysis was used to construct a prediction model for the outcome of ECV and covariates parity, maternal age, gestational age at ECV, fetal weight at ECV and AFI at ECV (Table 3). A parity ≥ 1 and a higher maternal age were found to be favorable predictors of successful ECV in our prediction model.

## 4. Discussions

This study was performed in a clinic where the CS rate is lower than the reported CS rate for Germany, which is about 31.8% according to the official statistics [3]. In 2000, a large international multicenter randomized clinical trial, called the Term Breech Trial, compared vaginal deliveries with planned cesarean deliveries [21]. It was shown that perinatal and neonatal mortality rates, as well as serious neonatal morbidity rates, were significantly higher in the planned vaginal delivery group than in the planned cesarean delivery group (16% vs. 5%) at breech presentation. These findings significantly led to obstetricians choosing CS as the safer option for breech delivery in the 2000s [9]. For this reason, more than 12% of the CSs in Germany are performed in case of breech presentation. For example, in the west-central part of Germany, in the State of Hessen, about 90% of breech fetuses at term are delivered via CS [3]. In our clinic, CS at breech presentation represented 4.8% of all registered deliveries from 2016 to 2020. 

In case of fetal breech position, ECV could be a successful and safe option to reduce the number of CSs [22,23]. The routine use of ECV could lower the rate of surgical delivery in case of breech presentation by approximately two-thirds in term pregnancies [9]. In most cases, fenoterol is used as tocolytic therapy, mainly as a continuous tocolysis. The improvement of the monitoring during the ECV with sonography and CTG and the use of tocolytic therapy made this method safer, thus reducing the complication rate associated with ECV [18]. 

By performing ECV, we aim to increase the proportion of vaginal cephalic delivery and thereby decrease the rate of CSs. For these reasons, ECV can be considered the first-line management in dealing with uncomplicated breech presentation at term. The method is recommended by Cochrane and the American and Royal Colleges of Obstetrics and Gynecologists, as well as by the German Society for Gynecology and Obstetrics (Deutsche Gesellschaft für Gynäkologie und Geburtshilfe) [3,24,25].

ECV would be generally recommended after 37 weeks of gestation [9,16]. It is performed as an elective procedure in non-laboring women, aiming to improve the chance of vaginal cephalic birth. Attempting ECV before term, between 34th and 36th pregnancy weeks, can be associated with an increase in late preterm birth [17]. According to the German guidelines, ECV should be offered to all women with uncomplicated breech presentation by singleton pregnancies in hospitals where facilities for an emergency CS are present [3,20]. In a study performed by Weiniger et al., the CS rate among women with successful ECV was 20.2%, whereas among women with persistent breech presentation at delivery it was 94.9% [26]. We registered a CS rate for successful ECV of 19.4%, while the unsuccessful ECV patients delivered through CS. 

Furthermore, women who underwent vaginal delivery after a successful ECV had lower odds of developing endometriosis and sepsis and shorter hospitalization, therefore lower hospital charges [26]. In contrast, these women could have a higher risk of chorioamnionitis. Attempted ECV may be also associated with an increased risk of a low APGAR score at 5 min [6]. According to the literature, the absolute risk of all complications of ECV is approximately 1% in fetuses at term [14]. We noticed in our study that the registered complications were minimal and insignificant compared to the high rate of successful ECV, followed by a high rate of vaginal deliveries. 

Women with singleton pregnancy and breech presented fetus without the following pathologies are potentially eligible for ECV near term (≥36 weeks). These pathologies include multiple gestation, onset of active labor, rupture of membranes, oligohydramnios, antepartum hemorrhage or history with placental abruption, pelvic abnormalities, severe preeclampsia or eclampsia, pathological Doppler or CTG, placenta praevia, placenta accreta, and infant with major congenital anomalies or growth restrictions [2].A point system, such as Kainer score, can be helpful to estimate the success rate of ECV, which includes parameters, such as AFI, placental location, fetal position, nuchal cord, estimated fetal weight, parity, fetal engagement, and uterine tone [27,28]. We noticed positive results even though we did not apply this score.

Multiparous women are known to have higher ECV success rates [9]. Our study shows that multigravidity and a parity ≥ 1 are associated with successful ECV. The absence of nulliparity was also identified as an important predictor of successful ECV, which supports the findings of previous studies.

According to the literature, ECV is considered safe in women with a history of CS and some studies showed that the success rate of ECV is comparable to that of women with no previous CS [29,30,31,32]. Although rare, we registered four cases with a history of CS. ECV was successful in three of them, but only one delivered vaginally. In our sample, the fetal back faced either the maternal left or right. We found no statistically significant relationship between the fetal position and the outcome of the maneuver. 

The anterior placental location has been reported as being associated with a lower rate of success, probably due to the anterior location of the placenta making it difficult to perform ECV [9]. In the present study, we included patients with anterior, posterior, lateral, and fundal placental location. We noticed that the relationship between placental location and ECV outcome was not significant.

Our study included women between 18 and 43 years old. The group with successful ECV had a higher mean maternal age than the group with unsuccessful ECV, therefore we included maternal age in our logistic regression analysis. In our prediction model, higher maternal age was found to be a predictor for successful ECV, therefore the success rate increases with maternal age. Other studies did report similar results [33,34]. It is important to note that there may be other related variables affecting this relationship, for example, BMI, which we did not take into account. According to the literature, high BMI values are associated with a low success rate of ECV and a decrease in the rate of vaginal delivery after successful ECV [35]. 

The relationship between estimated fetal weight at ECV and ECV outcome is controversial [9,34]. We found an association between the success of the maneuver and higher fetal weight, as well as higher gestational age at ECV. An explanation could be that a larger fetus, which corresponds to a higher gestational age, is more palpable [27,36]. 

It has been reported that a higher AFI is associated with successful ECV [18,37,38]. In the present study, the group with successful ECV had a higher mean AFI than the group with unsuccessful ECV. It is important to note that the minimum AFI score registered was eight.

The safety, efficacy, and cost-effectiveness of ECV for breech presentation followed by vaginal delivery are underlined in our study through good clinical practice and are sustained by other performed studies [2].

## 5. Conclusions

ECV for breech presentation is a safe procedure with a good success rate which increases the proportion of vaginal births. Maternal and fetal parameters can be used to estimate the chances of successful ECV. Multigravidity, absence of nulliparity, higher maternal age, higher gestational age, higher fetal weight, and higher AFI are all associated with successful ECV.

## Figures and Tables

**Table 1:** Association between outcome ECV and gravidity, parity, history of CS, fetal back position before ECV and placental location.

Variables	Successful ECV	Unsuccessful ECV	Test Statistic,
	(*n* = 62)	(*n* = 51)	*p*-Value
Gravidity			*χ*^2^(1) = 17.11, *p* < 0.001
primigravida	22 (35.5%)	38 (74.5%)	
multigravida	40 (64.5%)	13 (25.5%)	
Parity			*χ*^2^(1) = 19.73, *p* < 0.001
nullipara	27 (43.5%)	43 (84.3%)	
parity ≥ 1	35 (56.5%)	8 (15.7%)	
History of CS			Fisher’s exact, *p* = 0.63
no	59 (95.2%)	50 (98.0%)	
yes	3 (4.8%)	1 (2.0%)	
Fetal back position			*χ*^2^(1) = 3.47, *p* = 0.06
left	28 (45.2%)	32 (62.7%)	
right	34 (54.8%)	19 (37.3%)	
Placental location:			*χ*^2^(1) = 1.52, *p* = 0.22
anterior	29 (46.8%)	18 (35.3%)	
posterior or lateral	33 (53.2%)	33 (64.7%)	

**Table 2:** Comparison between maternal age, gestational age at ECV, fetal weight at ECV and AFI at ECV for successful and unsuccessful ECV using independent samples *t*-test.

Variables	Successful ECV (*n* = 62)	Unsuccessful ECV(*n* = 51)	Test Statistic, *p*-Value
Maternal age	32.84 (*SD* = 3.81)	30.29 (*SD* = 4.77)	*t*(111) = 3.15, *p* = 0.002
Gestational age at ECV	262.84 (*SD* = 6.15)	260.59 (*SD* = 2.59)	*t*(85.26) = 2.62, *p* = 0.01
Fetal weight at ECV	3106.10 (*SD* = 371.37)	2795.73 (*SD* = 346.98)	*t*(111) = 4.55, *p* < 0.001
AFI at ECV	15.56 (*SD* = 3.60)	14.06 (*SD* = 3.42)	*t*(111) = 2.26, *p* = 0.03

**Table 3:** Results of multiple logistic regression analysis for predictors of successful ECV.

Predictors	OR (Odds Ratio)	95% CI (Confidence Interval)	*p*-Value
Parity ≥ 1	3.570	1.299–9.807	0.014
Maternal age	1.133	1.016–1.262	0.025
Gestational age at ECV	1.105	0.971–1.259	0.131
Fetal weight at ECV	1.001	0.999–1.003	0.066
AFI at ECV	1.059	0.913–1.229	0.449
